# Yeast Gis2 and Its Human Ortholog CNBP Are Novel Components of Stress-Induced RNP Granules

**DOI:** 10.1371/journal.pone.0052824

**Published:** 2012-12-21

**Authors:** Marta Rojas, George W. Farr, Cesar F. Fernandez, Laura Lauden, John C. McCormack, Sandra L. Wolin

**Affiliations:** 1 Department of Cell Biology, Yale University School of Medicine, New Haven, Connecticut, United States of America; 2 Department of Genetics and Howard Hughes Medical Institute, Yale University School of Medicine, New Haven, Connecticut, United States of America; German Cancer Research Center, Germany

## Abstract

Although a CCTG expansion in the gene encoding the zinc knuckle protein CNBP causes a common form of muscular dystrophy, the function of both human CNBP and its putative budding yeast ortholog Gis2 remain poorly understood. Here we report the protein interactions of Gis2 and the subcellular locations of both Gis2 and CNBP. We found that Gis2 exhibits RNA-dependent interactions with two proteins involved in mRNA recognition, the poly(A) binding protein and the translation initiation factor eIF4G. We show that Gis2 is a component of two large RNA-protein granules, processing bodies and stress granules, which contain translationally repressed mRNAs. Consistent with a functional ortholog, CNBP also associates with the poly(A) binding protein and accumulates in stress granules during arsenite treatment of human cells. These results implicate both Gis2 and CNBP in mRNA handling during stress.

## Introduction

The numerous conserved RNA-binding proteins in eukaryotic cells influence the metabolism, structure and function of their target RNAs in diverse ways. The importance of these proteins for normal cell physiology is underscored by the increasing realization that defects in RNA-binding proteins underlie a range of human diseases [1]–[3]. Nonetheless, despite the progress that has been made in characterizing RNA-binding proteins, grouping them into families based on their structural domains, and identifying their RNA targets and cellular roles, the functions of many conserved and clinically important RNA-binding proteins remain poorly understood.

One such RNA-binding protein is the cellular nucleic acid binding protein CNBP (also called ZNF9, zinc finger nine). A CCTG repeat expansion in the *CNBP* first intron causes the autosomal dominant disease myotonic dystrophy type 2 (DM2) [1]. The presence of CCUG repeats in the CNBP pre-mRNA contribute to DM2 by sequestering the RNA-binding proteins MBNL1 (muscleblind-like 1) and CUGBP1 (CUG-binding protein 1) [4]. Although studies initially reported that CNBP levels were unaffected in cells and tissues from DM2 patients [5], [6], other laboratories have found that CNBP protein and RNA levels are reduced in patient specimens [7]–[9]. Intriguingly, mice in which one *CNBP* allele is inactivated display features of DM2, including myotonia and muscle wasting [10], suggesting that decreased CNBP could contribute to the disease. In support of a key cellular role, CNBP is essential for mouse development [11], and likely orthologs exist in many animal species and in fungi [12]–[15].

Despite its potential importance and conservation, the function of CNBP remains poorly understood. CNBP is 18.7 kDa and consists largely of seven CCHC zinc knuckles (CX_2_CX_4_HX_4_C; C = Cys, H = His, X = any amino acid). Structural studies of similar zinc knuckles in retroviral nucleocapsid proteins and the Air2 subunit of the *S. cerevisiae* TRAMP poly(A) polymerase have revealed that they interact with single-stranded RNA [16] and can also be protein-protein interaction modules [17]. CNBP has been described to bind both single-stranded DNA and RNA, and biochemical assays have suggested roles for CNBP in numerous processes, including transcriptional regulation, translation and internal initiation of translation [7], [9], [18]–[25].

Similar to the mammalian protein, the roles of the fission and budding yeast CNBP orthologs remain under investigation. *S. pombe* Byr3, which is required for efficient conjugation of fission yeast, has been reported to both bind double-stranded DNA and to co-purify with the Dicer ribonuclease [12], [26]. *S. cerevisiae GIS2* (GIG Suppressor), which was discovered in a screen for high copy suppressors of a strain unable to grow in galactose [13], was reported to sediment with polyribosomes in yeast extracts and to substitute for CNBP in stimulating cap-independent translation in human cells [15]. Recently, using a combination of microarray experiments and proteomics, Gis2 was reported to interact with motifs in the coding sequences of hundreds of mRNAs and coordinate the expression of these mRNAs as part of an “RNA regulon” [27].

Because elucidation of the roles of CNBP and its orthologs could be helpful for understanding DM2 pathogenesis, we examined the protein interactions and subcellular location of *S. cerevisiae* Gis2. We report that Gis2 exhibits RNA-dependent interactions with the translation initiation factor eIF4G and the poly(A) binding protein Pab1. We identify Gis2 as a novel component of two cytoplasmic structures containing translationally repressed mRNPs, P-bodies and stress granules. Consistent with a functional ortholog, we show that CNBP also associates with the cytoplasmic poly(A) binding protein and localizes to stress granules upon arsenite treatment of human cells. Our data are consistent with a model in which both Gis2 and CNBP participate in mRNA handling during stress.

## Results

### Gis2 Interacts with Components Involved in mRNA Translation

**Figure 1:**
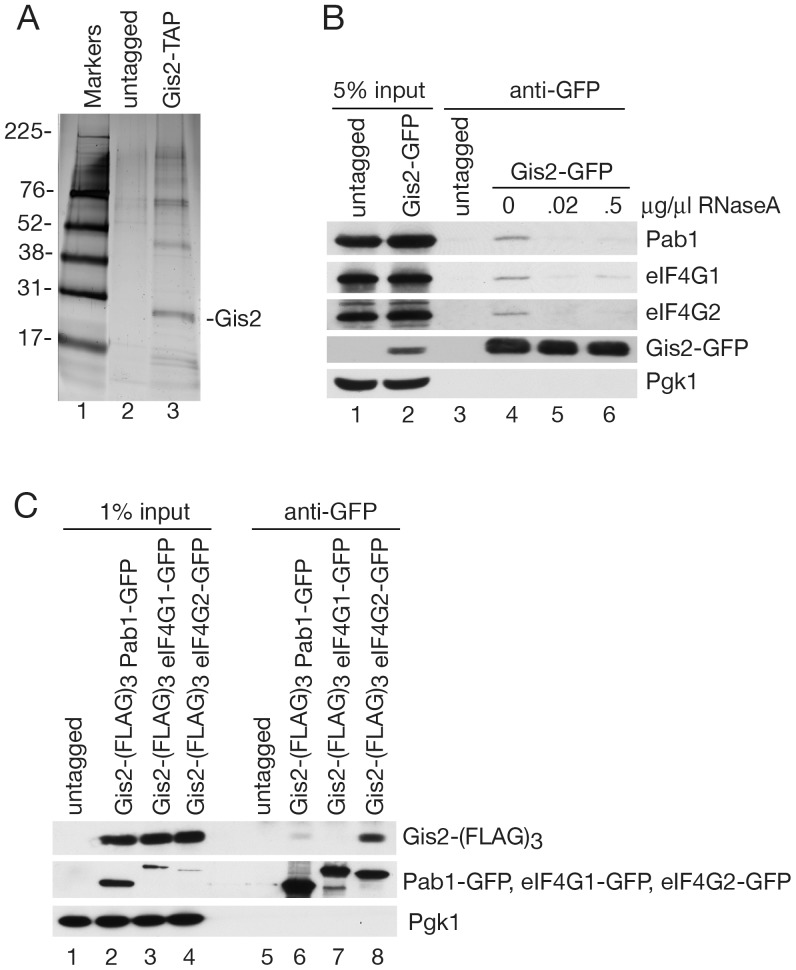
Gis2 associates with proteins involved in translation initiation. (A) After tandem affinity purification, eluates from an untagged strain and a strain expressing Gis2-TAP were fractionated in a SDS-polyacrylamide gel and proteins visualized by silver staining. Lane 1, molecular size markers. Sizes are in kDa. (B) Lysates of an untagged strain and a strain expressing Gis2-GFP were subjected to immunoprecipitation with anti-GFP antibodies. Prior to immunoprecipitation, Gis2-GFP lysates were incubated with the indicated amounts of RNase A. Proteins in immunoprecipitates were detected by Western blotting with antibodies against Pab1, eIF4G1, and eIF4G2. The efficiency of immunoprecipitation was determined by re-probing with anti-GFP. As a negative control, the blot was reprobed to detect Pgk1. (C) Lysates of untagged and *Gis2-(FLAG*)*_3_* strains expressing Pab1-GFP, eIF4G1-GFP or eIF4G2-GFP were subjected to immunoprecipitation with anti-GFP antibodies. After Western blotting, Gis2-(FLAG)_3_ was detected with anti-FLAG antibodies. To examine immunoprecipitation efficiency, Pab1-GFP, eIF4G1-GFP and eIF4G2-GFP were detected with anti-GFP antibodies. Pgk1 was detected as a negative control.

To identify Gis2-associated proteins, we subjected a strain in which Gis2 was fused to a TAP module to two rounds of affinity purification. Silver staining of the final eluate revealed Gis2 and several bands that were not detected in a parallel purification from an untagged strain (Figure 1A). Proteins in both eluates were analyzed using multidimensional protein identification technology (MUDPIT) [28]. After filtering out proteins that are common contaminants of TAP purifications [29], the most abundant proteins in the Gis2-TAP eluate included the poly(A) binding protein Pab1, the two isoforms of the translation initiation factor eIF4G (eIF4G1 and eIF4G2) and numerous ribosomal proteins (Table S1). Several other proteins were also linked to translation initiation, such as the cap-binding protein eIF4E [30], or mRNA stability, such as Xrn1, the major 5′ to 3′ exoribonuclease that carries out mRNA decay [31].

To validate the interactions, we focused on Pab1, eIF4G1 and eIF4G2. Pab1 and eIF4G, together with eIF4E and eIF4A, are involved in cap-dependent translation initiation [32]. Specifically, eIF4G, together with eIF4E and the DExD/H helicase eIF4A, forms the cap-binding complex eIF4F. Association of eIF4G with Pab1, which binds the mRNA poly(A) tail, circularizes the mRNA and increases the efficiency of recruiting 43S initiation complexes [32], [33]. Using anti-GFP antibodies to immunoprecipitate from *GIS2-GFP* cell lysates, followed by Western blotting of proteins in immunoprecipitates, we confirmed that a small fraction of Pab1, eIF4G1 and eIF4G2 associates with Gis2-GFP (Figure 1B, lane 4). Treatment of the lysates with RNase A revealed that the interaction of Gis2-GFP with all three proteins depends on RNA (Figure 1B, lanes 5–6). Although the amounts of Pab1, eIF4G1 and eIF4G2 associated with Gis2-GFP was low, these proteins were not detected in an immunoprecipitate from an untagged strain (lane 3). Additionally, reprobing of the blot with antibodies to 3-phosphoglycerate kinase, which is among the most abundant *S. cerevisiae* proteins [34], failed to detect this protein in immunoprecipitates (Figure 1B). Finally, although the Ded1 ATPase, which interacts with eIF4G [35], was an abundant component of the Gis2-TAP eluate (Table S1), we also failed to detect this protein in our immunoprecipitates (data not shown), consistent with reports that it is a frequent contaminant of tandem affinity purifications [29].

We confirmed the interactions between Gis2, Pab1 and eIF4G by using anti-GFP antibodies to immunoprecipitate from strains that carried Pab1-GFP, eIF4G1-GFP or eIF4G2-GFP and also contained Gis2 fused to 3 copies of FLAG. Western blotting with anti-FLAG revealed Gis2-(FLAG)_3_ in both the Pab1-GFP and eIF4G2-GFP immunoprecipitates (Figure 1C), but did not detect this protein in the eIF4G1-GFP immunoprecipitate. We conclude that a small fraction of Gis2 associates with both Pab1 and eIF4G2 and possibly also eIF4G1, and that this interaction requires RNA.

### A Small Fraction of Gis2 may Associate with Polyribosomes

**Figure 2:**
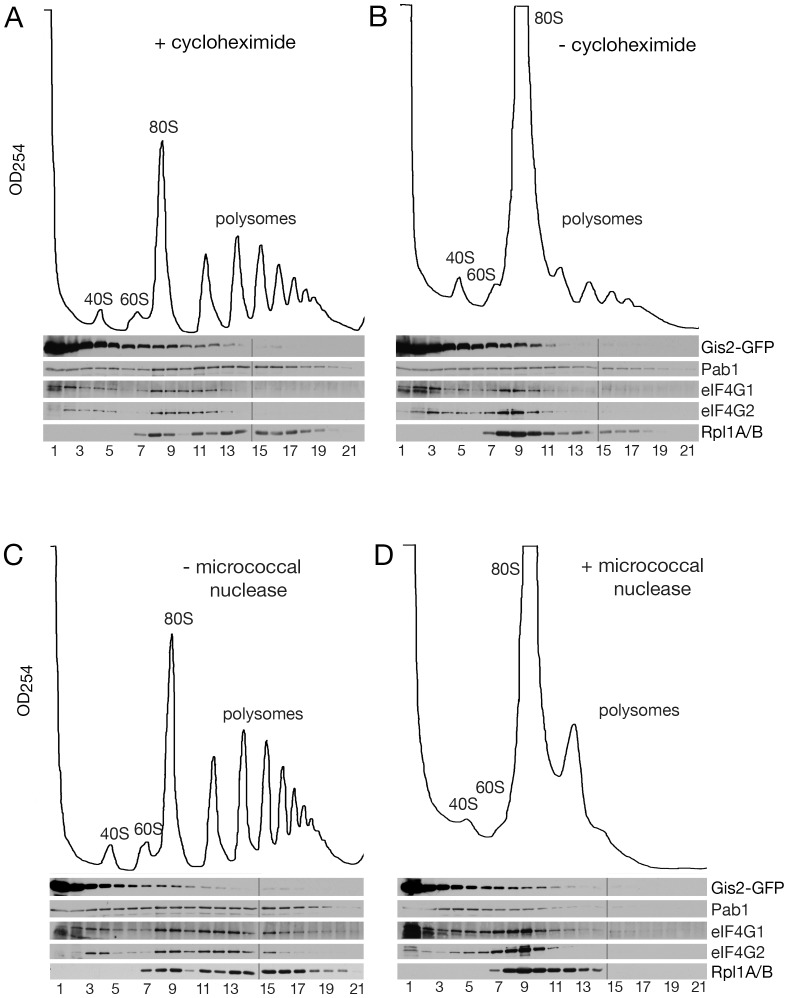
A small fraction of Gis2 sediments with polyribosomes. (A and B) *GIS2-GFP* cell lysates were prepared in the presence (A) or absence (B) of cycloheximide and fractionated in 15–50% sucrose gradients. Fractions were collected while monitoring OD_254_. Proteins were subjected to Western blotting to detect Gis2-GFP, Pab1, eIF4G1, eIF4G2 and ribosomal proteins L1A and L1B. (C and D) *GIS2-GFP* cell lysates prepared in the presence of cycloheximide were either untreated (C) or incubated with 5 U/µl micrococcal nuclease (D) prior to sedimentation. Fractions from each gradient were analyzed in two gels as indicated by the lines.

The large number of proteins from the small and large ribosomal subunits in our Gis2-TAP purification (Table S1), coupled with a report that Gis2 sediments with polyribosomes [15], prompted us to examine whether Gis2 was polyribosome-associated. *GIS2-GFP* lysates were prepared in the presence of cycloheximide, which stabilizes translating ribosomes, and subjected to sucrose gradient sedimentation (Figure 2A). Western blotting revealed that most Gis2-GFP sedimented at the top of the gradient (fractions 1–3; 55.8%). However, some Gis2-GFP sedimented in fractions containing ribosomal subunits and monoribosomes (fractions 4–10; 39.7%), and a small amount was found in polyribosome-containing fractions (fractions 11–21; 4.5%). Reprobing to detect Pab1 revealed that this protein was found throughout the gradient, as described [36], [37].

To determine if the Gis2-GFP that sedimented with polyribosomes was indeed polyribosome-associated, we disrupted polyribosomes before performing gradient fractionation. Experiments in which we omitted the cycloheximide resulted in decreased polyribosomes, with a concomitant increase in 80S monoribosomes (Figure 2B). Treatment of the lysate with micrococcal nuclease to degrade portions of mRNA that are not protected by ribosomes also converted most polysomes to 80S monosomes (Figure 2D). Western blotting to detect the large ribosomal subunit proteins Rpl1A and Rpl1B confirmed that both treatments were effective at disrupting polyribosomes (Figures 2B and 2D). Following both treatments, the amount of Gis2-GFP present in polyribosome-containing fractions (fractions 11–21) was reduced (Figures 2B and 2D) (to 1.3% and 1.2%, respectively). Thus, a small fraction of Gis2-GFP may be polyribosome-associated.

### Gis2 Accumulates in P-bodies and Stress Granules during Glucose Deprivation and Growth in Stationary Phase

Pab1, eIF4G1 and eIF4G2 are all components of stress granules (also called EGP bodies), cytoplasmic mRNA-containing granules that form when translation initiation is impaired [37]–[40]. We therefore examined whether Gis2 was a component of stress granules or related structures called processing bodies (P-bodies) that share some components with stress granules, but also contain components of the mRNA decapping and 5' to 3' decay machinery [39], [41]. Strains in which the chromosomal *GIS2* was fused to mCherry (mCh) were used, together with P-body and stress granule markers fused to GFP, to localize Gis2 following stresses that cause accumulation of these RNP granules.

**Figure 3:**
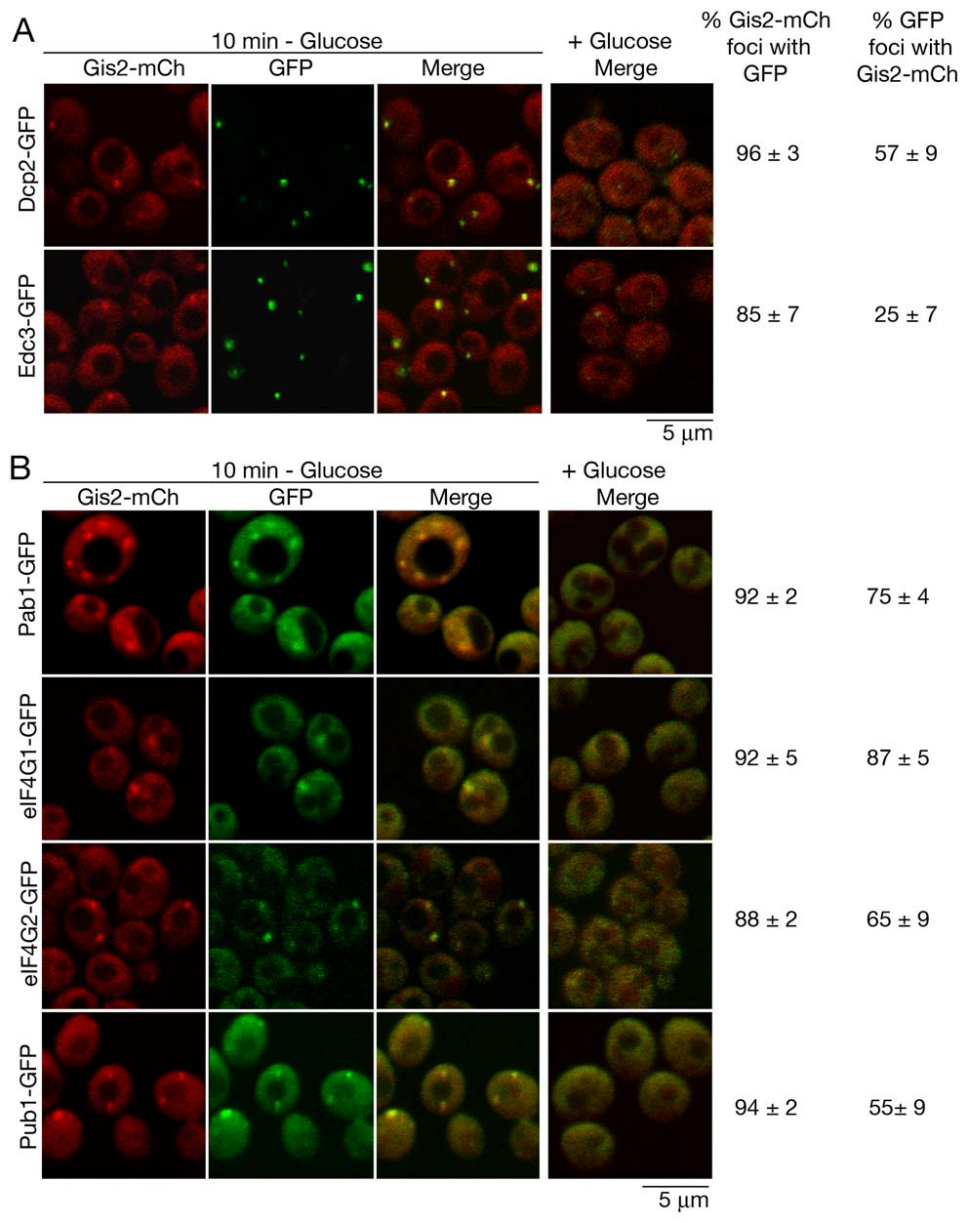
Gis2 accumulates in P-bodies and stress granules during glucose depletion. (A and B) Yeast strains expressing chromosomal Gis2-mCh and (A) the P-body markers Dcp2-GFP and Edc3-GFP or (B) the stress granule markers Pab1-GFP, eIF4G1-GFP, eIF4G2-GFP and Pub1-GFP were grown in glucose-containing media, then resuspended in fresh media that either lacked or contained glucose. After 10 minutes, cells were observed using confocal microscopy. In glucose media (right column), no Gis2-mCh foci were observed; thus only the merged panels are shown. Bars, 5 µm.

First, we examined the effects of glucose deprivation on Gis2 localization. Both P-bodies and stress granules become prominent when yeast cells are shifted to media lacking glucose for 10–30 min [37], [38], [42]. Although Gis2-mCh showed homogeneous cytoplasmic staining during logarithmic growth in rich media, a fraction localized to discrete cytoplasmic granules following 10 min of glucose deprivation (Figure 3A). Examination of two P-body markers, Dcp2, a subunit of the mRNA decapping enzyme, and Edc3, an enhancer of decapping [43], revealed that most Gis2 foci localized with GFP-tagged forms of these proteins (Figure 3A). However, as only 57% of the Dcp2-GFP foci and 25% of the Edc3-GFP foci co-localized with Gis2-mCh, many P-bodies do not contain Gis2-mCh. Most Gis2-mCh foci also co-localized with the stress granule markers Pab1-GFP, eIF4G1-GFP, eIF4G2-GFP and Pub1-GFP (Figure 3B), consistent with reports that stress granule and P-body markers are often found in the same foci in budding yeast [37], [44]. Notably, most Pab1-GFP (75%), eIF4G1-GFP (87%) and eIF4G2-GFP (65%) foci also contained Gis2-mCh. Somewhat less co-localization was seen with Pub1, a protein that exhibits homology to the mammalian stress granule marker TIA-1 [38]. Only 55% of Pub1-GFP foci also contained Gis2-mCh, consistent with previously described heterogeneity in stress granule composition [40].

**Figure 4:**
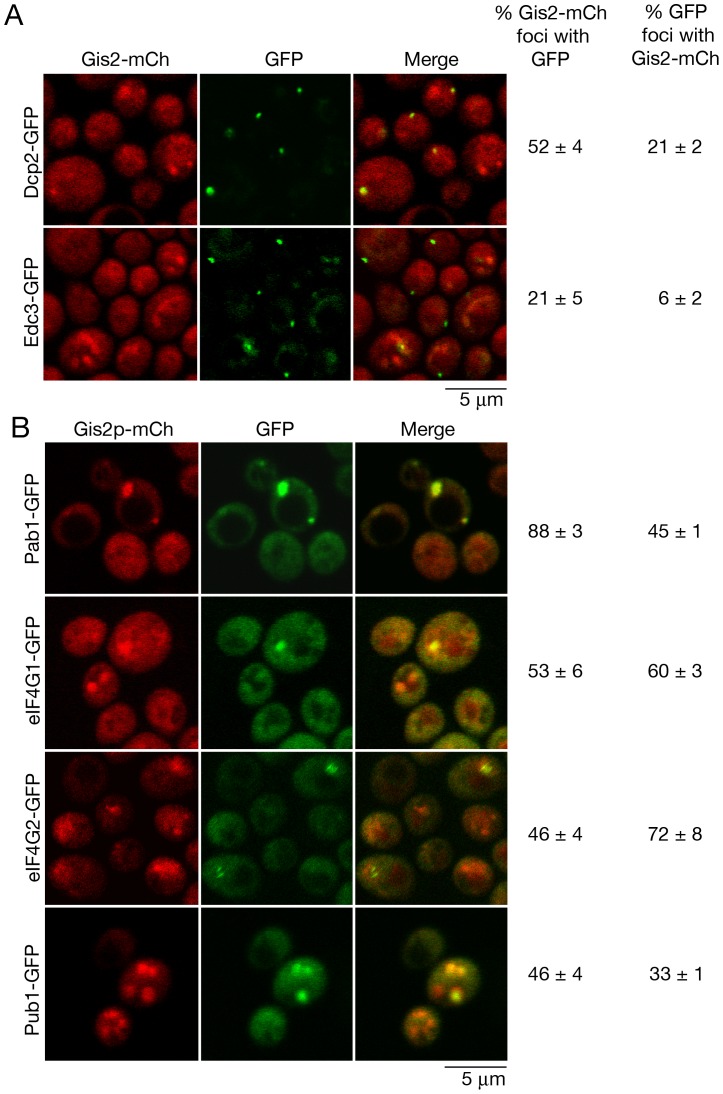
Gis2 accumulates in P-bodies and stress granules during stationary phase. (A and B) Yeast strains expressing chromosomal Gis2-mCh and the indicated (A) P-body or (B) stress granule markers were grown for 4 days in glucose-containing media and examined using confocal microscopy. Scale bar, 5 µm.

P-bodies and stress granules also accumulate in stationary phase [44]. As expected for a component of one or both bodies, Gis2-mCh localized to cytoplasmic foci during growth in stationary phase (Figure 4). Co-localization experiments revealed that although 52% of the Gis2-mCh foci co-localized with the P-body marker Dcp2-GFP and 21% with Edc3-GFP, only 21% of Dcp2-GFP and 6% of Edc3-GFP containing foci also contained Gis2-mCh. Thus, in stationary phase, most P-bodies do not contain Gis2-mCh (Figure 4A). Examination of stress granule markers revealed that these proteins also partly co-localized with Gis2-mCh, with the strongest co-localization (88%) observed with Pab1-GFP. However, as 45% of Pab1-GFP and 33% of Pub1-GFP foci co-localized with Gis2-mCh, only a subset of stress granules contains Gis2-mCh (Figure 4B).

To determine if Gis2 was important for formation of P-bodies or stress granules, we compared the accumulation of these structures in wild-type and *gis2*Δ cells carrying plasmids that express Dcp2 fused to red fluorescent protein (RFP) and Pub1-mCh. No significant differences were identified in either the number or size of these bodies in *gis2*Δ cells (data not shown). Additionally, experiments in which we used anti-GFP antibodies to immunoprecipitate from *GIS2-GFP* cells during glucose deprivation and stationary phase did not reveal reproducible increases in the association of eIF4G1, eIF4G2 or Pab1 with Gis2-GFP under either of these stress conditions (data not shown).

### Examination of Translational Repression and mRNA Decay in *gis2*Δ Cells

**Figure 5:**
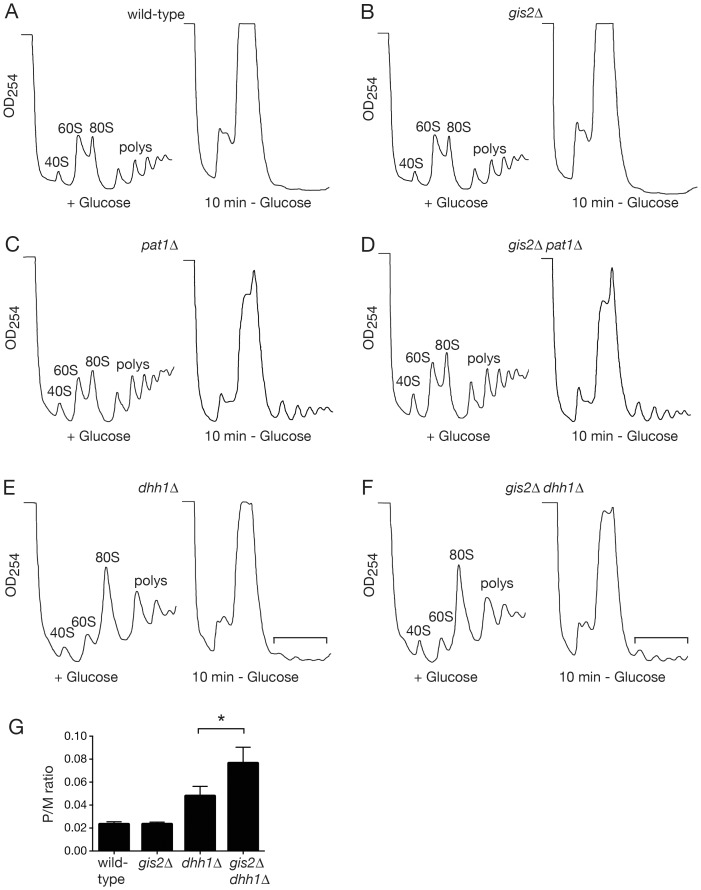
Polysome profiles following glucose deprivation of yeast cells. (A–F) Wild-type and the indicated mutant strains were grown in glucose-containing media until early logarithmic phase, pelleted, and resuspended in glucose-containing media (left panels) or in media lacking glucose (right panels) and grown for an additional 10 minutes. Lysates were fractionated in 15–50% sucrose gradients and the positions of ribosomal subunits, monoribosomes and polyribosomes detected by monitoring OD_254_ during collection. (A) wild-type, (B) *gis2*Δ , (C) *pat1*Δ, (D) *gis2*Δ*pat1*Δ, (E) *dhh1*Δ, (F) *gis2*Δ*dhh1*Δ cells. To ensure reproducibility, each mutant was analyzed at least twice. (G) The P/M ratio was determined for wild-type, *gis2*Δ, *dhh1*Δ and *gis2*Δ *dhh1*Δ strains as described [63] following 10 minutes of glucose depletion. P/M ratios for wild-type and *gis2*Δ strains were determined from three biological replicates, while the P/M ratios for *dhh1*Δ and *dhh1*Δ *gis2*Δ strains were determined from four replicates. Asterisk, p<.05, two-tailed paired t-test.

Since many P-body and stress granule components function in translational repression and/or mRNA decay [39], [40], we examined whether Gis2 contributes to these processes. A well-studied example of both translational repression and mRNA decay occurs when yeast growing in glucose-containing media are incubated in media lacking glucose [45]–[47]. Within 10 min, polyribosomes are greatly reduced and there is a concomitant spike in 80S ribosomes [45], [46] (also Figure 5A). Two proteins, the DEAD box helicase Dhh1 and the decapping activator Pat1, function in parallel pathways to repress translation during glucose deprivation [46], [48]. Examination of *gis2*Δ yeast revealed that translational repression was similar to wild-type cells (Figure 5B). Since neither *pat1*Δ nor *dhh1*Δ cells fully repress translation upon glucose deprivation [46], we examined whether Gis2 affected translational repression in these mutants. Although translational repression in *gis2*Δ *pat1*Δ cells was similar to *pat1*Δ cells (Figures 5C and 5D), we observed a small but reproducible enhancement in the polyribosome pool when *gis2*Δ *dhh1*Δ cells were compared with *dhh1*Δ cells (Figures 5E and 5F, brackets). Quantitation of the polysome to monosome (P/M) ratio for multiple experiments revealed that although the P/M ratio of *gis2*Δ lysates upon glucose depletion was indistinguishable from wild-type lysates, the P/M ratio for *dhh1*Δ lysates was 2.1-fold (±.4) higher than for wild-type lysates, while *gis2*Δ *dhh1*Δ lysates had a P/M ratio that was 3.3-fold (±.6) higher than wild-type and *gis2*Δ lysates (Figure 5G), suggesting that Gis2 may contribute to translational repression in *dhh1*Δ cells.

Because ^35^S-methionine incorporation in wild-type cells is strongly inhibited upon glucose deprivation [45], but is still detectable in *dhh1*Δ mutants [49], we examined whether we could detect enhanced incorporation in *gis2*Δ *dhh1*Δ cells. As expected from the polyribosome profiles, ^35^S-methionine incorporation was almost completely inhibited in wild-type and *gis2*Δ cells (reduced by 97.6±1.9% and 98.1±1.1%, respectively) following glucose deprivation (Figure S1). Consistent with the small increase in polyribosomes in *gis2*Δ *dhh1*Δ cells, the rate of ^35^S-methionine incorporation was always slightly higher in *gis2*Δ *dhh1*Δ cells than in *dhh1*Δ cells following glucose removal. However, the difference did not reach statistical significance, with ^35^S-methionine incorporation reduced by 91.1±2.0% in *dhh1*Δ and 88.1±2.2% in *gis2*Δ *dhh1*Δ cells (Figure S1), possibly because the already low levels of translation in *dhh1*Δ cells during glucose deprivation made it difficult to document small changes in translation efficiency. Nonetheless, the small but reproducible increase in polysomes detected in *gis2*Δ *dhh1*Δ cells compared to *dhh1*Δ cells during glucose deprivation (Figures 5E and 5F) suggests that Gis2 could contribute to translational repression of at least some mRNAs.

We also examined whether Gis2 has a general role in mRNA decay. For these experiments, two mRNA reporters, *PGK1pG* and *MFA2pG*, each under control of the *GAL1* promoter [50], were integrated into the genome of wild-type and *gis2*Δ cells at the *CUP1* locus. These reporters have been widely used to measure mRNA half-lives, by first growing yeast in galactose media to allow expression of the reporters, then repressing transcription with glucose-containing media [51]. Both reporters exhibited similar decay rates in wild-type and *gis2*Δ cells (Figures S2A and S2B). We also used a similar reporter to detect *EDC1* mRNA, since decay of this mRNA is strongly impaired in *dhh1*Δ cells [52]. Decay of *EDC1* mRNA was unaffected in *gis2*Δ cells (Figure S2C). Moreover, although *EDC1* mRNA decay was slowed in *dhh1*Δ cells compared to wild-type cells, the decay rate in *gis2*Δ *dhh1*Δ cells was similar to that in *dhh1*Δ cells (Figure S2D). We conclude that Gis2 is not required for general mRNA decay, although we cannot exclude the possibility that it is involved in the degradation of a subset of mRNAs.

### Some CNBP Associates with Translating Ribosomes in Human Cells

**Figure 6:**
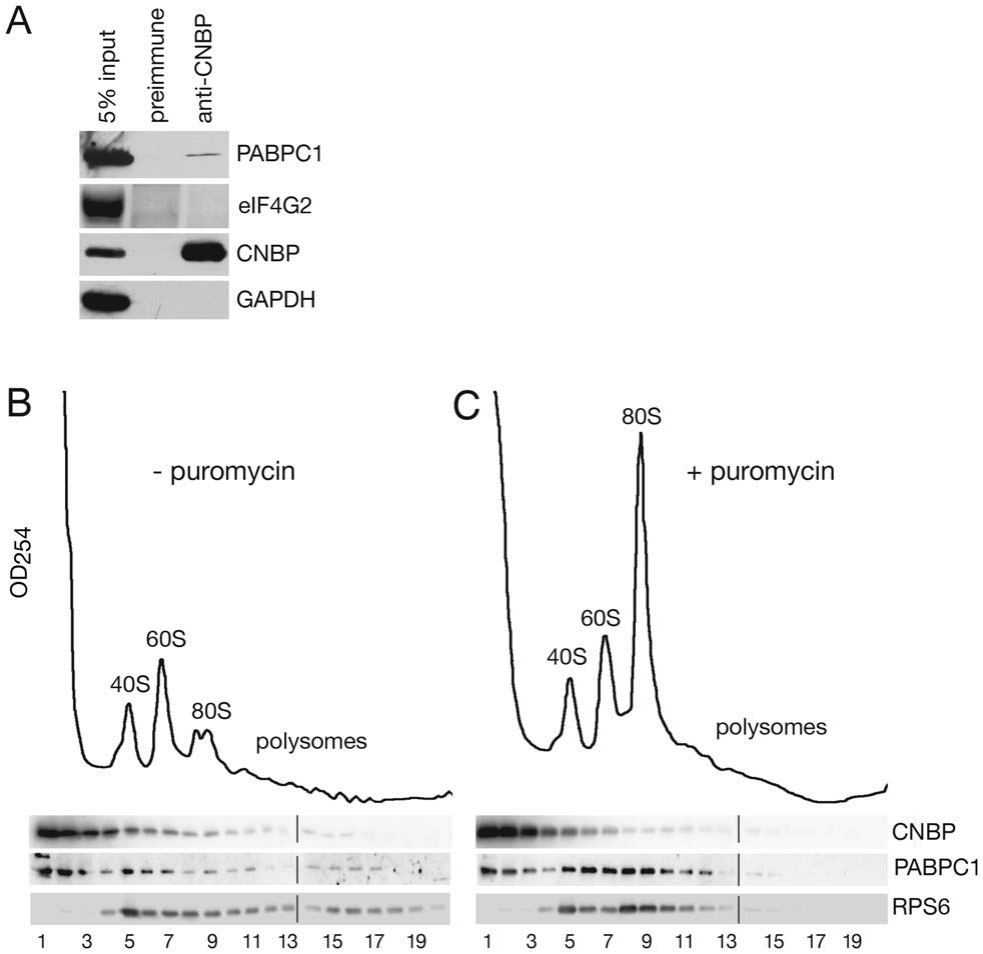
Some CNBP associates with PABPC1 and sediments with translating ribosomes. (A) HeLa cell lysates were subjected to immunoprecipitation with anti-CNBP antibodies. Proteins in immunoprecipitates were subjected to Western blotting to detect the poly(A) binding protein PABPC1 and eIF4G2. To assess the efficiency of immunoprecipitation, the level of CNBP in the immunoprecipitate was also determined. As a negative control, the blot was reprobed to detect GAPDH. (B and C) HeLa cells were either untreated (B) or incubated with puromycin for 20 minutes (C) prior to harvesting in cycloheximide. Lysates were sedimented in 15–50% sucrose gradients and fractions collected while monitoring OD_254_. Proteins were subjected to Western blotting to detect CNBP, PABP1C and ribosomal protein RPS6.

To examine the extent to which human CNBP is functionally similar to Gis2, we determined whether CNBP associates with translation initiation factors and/or polysomes. Immunoprecipitations from human HeLa cells using antibodies to CNBP [53], followed by Western blotting of proteins in the immunoprecipitate, revealed that a small fraction of the cytoplasmic poly(A) binding protein I PABPC1 was associated with CNBP. The presence of PABPC1 in the immunoprecipitate was specific, as both eIF4G2 and glyceraldehyde 3-phosphate dehydrogenase were not detected (Figure 6A). Western blotting to determine if the major eIF4G isoform, eIF4G1, associated with CNBP gave inconclusive results due to nonspecific background signals (data not shown).

To determine if CNBP associates with translating ribosomes, we harvested HeLa cells in the presence of cycloheximide and subjected the resulting lysates to sucrose gradient sedimentation (Figure 6B). As observed for Gis2-GFP, most CNBP sedimented in the lightest fractions (fractions 1–3, 74.6%). Additionally, some CNBP sedimented in fractions containing ribosomal subunits and monosomes (fractions 4–9, 23.7%) and a small amount was detected in polysome-containing fractions (fractions 10–20, 1.6%). Because omitting cycloheximide did not significantly alter the polyribosome profile as measured by UV absorbance (data not shown), we incubated the cells with puromycin, which causes premature termination of translation, prior to harvesting in cycloheximide. Puromycin was effective at reducing translation, as measured by decreased polysomes and increased 80S subunits (Figure 6C). Notably, following puromycin treatment, the fraction of CNBP in the lightest gradient fractions increased to 84.1%, while the amount of CNBP that sedimented with ribosomal subunits and 80S monosomes decreased (14.8%), as did the fraction that sedimented with polyribosomes (0.6%). We conclude that a small fraction of CNBP associates with translating ribosomes.

### CNBP Accumulates in Stress Granules

**Figure 7:**
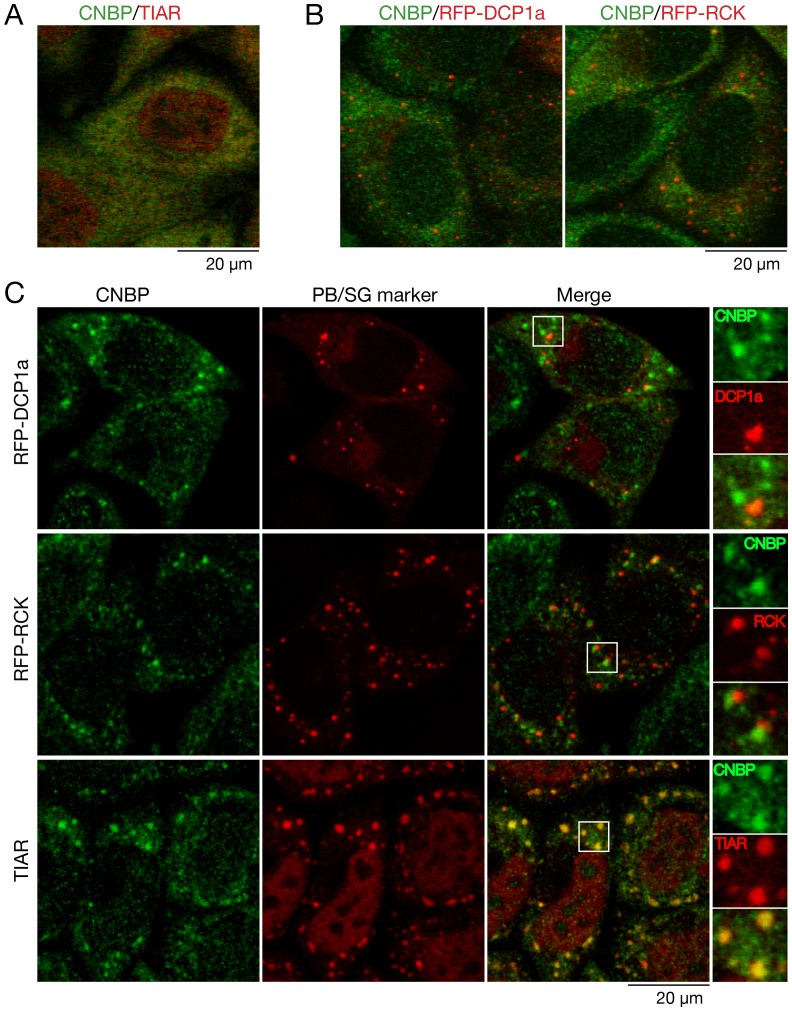
CNBP accumulates in stress granules during arsenite treatment of HeLa cells. (A) HeLa cells were subjected to immunofluorencence to detect CNBP (green) and the stress granule marker TIAR (red). A merged image is shown. Bar, 20 µm. (B) HeLa cells transfected with plasmids expressing RFP-DCP1a (left) or RFP-RCK (right) were subjected to immunofluorescence with anti-CNBP antibodies. Merged images are shown. Bar, 20 µm. (C) To induce P-bodies and stress granules, untransfected cells and cells expressing RFP-DCP1a or RFP-RCK were incubated with 500 µM arsenite for 30 minutes. Following immunofluorescence to detect CNBP (top and middle panels) or both CNBP and TIAR (bottom panel), cells were examined using confocal microscopy. Bar, 20 µm. The rightmost panels show enlarged images of the boxed areas.

Since our experiments revealed that Gis2 was a component of P-bodies and stress granules, we determined if this localization was conserved for CNBP. In contrast to yeast, mammalian stress granules and P-bodies exhibit far less overlap in their protein components [39], [40]. Using anti-CNBP antibodies in immunofluorescence experiments, we found that CNBP was mostly cytoplasmic in HeLa cells (Figure 7A). In these unstressed cells, immunofluorescence with an antibody to the stress granule marker TIAR revealed that this protein was concentrated in nuclei (Figure 7A), as described [54]. To both have P-body markers and to induce formation of small P-bodies, we transfected the HeLa cells with plasmids in which RFP was fused to either the Dcp1 ortholog DCP1a (RFP-DCP1a) [55] or the Dhh1 ortholog RCK (RFP-RCK). Although transfection of either plasmid resulted in P-body formation as described [55], [56], CNBP was not detected in these foci (Figure 7B).

To induce stress granules and increase P-body formation, we incubated the cells with arsenite, a strong inducer of oxidative stress. As expected [54], [55], both P-bodies and stress granules became prominent (Figure 7C). CNBP also accumulated in discrete cytoplasmic foci. Co-localization experiments revealed that the CNBP foci were largely distinct from P-bodies, as only 15±3% of the CNBP colocalized with RFP-DCP1a and 16±4% colocalized with RFP-RCK. In contrast, 93±7% of the CNBP foci also contained TIAR (Figure 7C). However, only 61±5% of the TIAR-containing foci contained CNBP, indicating the stress granules are heterogeneous in composition. Consistent with observations that arsenite-induced stress granules are often adjacent to P-bodies [55], many CNBP foci bordered P-bodies (Figure 7C, merge and insets).

**Figure 8:**
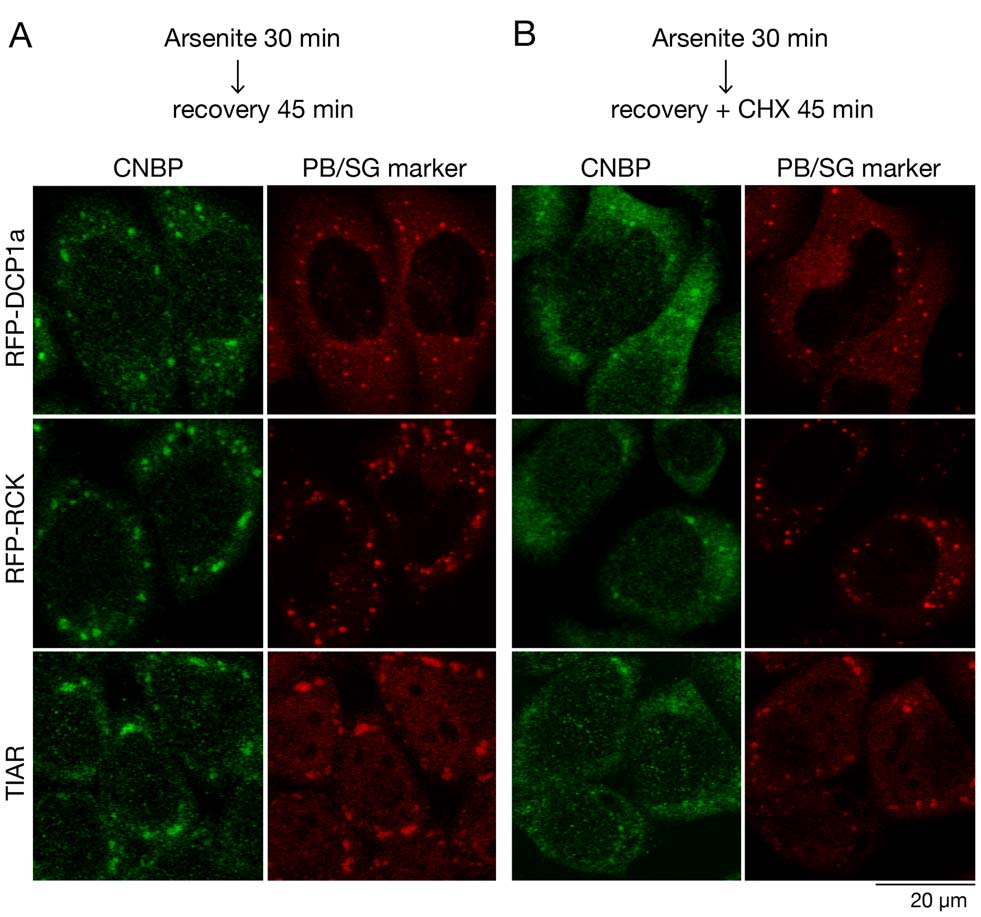
Cycloheximide enhances the rate of disappearance of CNBP-positive foci. (A and B) To induce P-bodies and stress granules, HeLa cells expressing RFP-DCP1a and RFP-RCK and untransfected cells were incubated with 500 µM arsenite for 30 minutes. Following arsenite treatment, cells were allowed to recover in fresh medium for 45 minutes in the (A) absence or (B) presence of cycloheximide. After fixation, transfected cells were subjected to immunofluorescence to detect CNBP (top and middle panels) or CNBP and TIAR (bottom panels) and examined using confocal microscopy.

Another way in which mammalian P-bodies and stress granules can be distinguished is by whether the presence of cycloheximide enhances their disassembly during recovery from arsenite [57]. Following arsenite removal, the rate of P-body disassembly is largely unaffected by cycloheximide, while stress granule disassembly increases [57], possibly because the mRNPs in stress granules continuously exchange with mRNPs entering polyribosomes [40], [57]. Thus, if the CNBP foci are stress granules, they should rapidly disassemble if cycloheximide is present in the culture medium during recovery from arsenite. As expected, the RFP-DCP1a and RFP-RCK foci were not strongly affected by cycloheximide (Figure 8). Quantitation revealed that RFP-DCP1a was detected in 21±1 foci per cell in the absence of cycloheximide and in 18±1 foci per cell when cycloheximide was present (14% decrease). Similarly, the RFP-RCK foci decreased from 19±1 without cycloheximide to 18±1 foci per cell in the presence of cycloheximide (5% decrease). In contrast, TIAR-containing foci were reduced by 58% (Figure 8) with 12±1 foci per cell detected in the absence of cycloheximide and 5±0 per cell when cycloheximide was present. Notably, the CNBP foci largely disassembled, as 9±0 per cell were detected in the absence of cycloheximide and 2±0 per cell when cycloheximide was present (78% decrease), although the few CNBP-positive foci that remained co-localized with TIAR (Figure 8).

We conclude that CNBP is a component of stress granules. The fact that CNBP is largely excluded from P-bodies, while Gis2 co-localizes with both P-body and stress granule markers, may reflect the fact that yeast P-bodies and stress granules exhibit more overlap with respect to protein composition than their mammalian counterparts [43].

### CNBP Depletion does not Affect Stress Granule Formation

**Figure 9:**
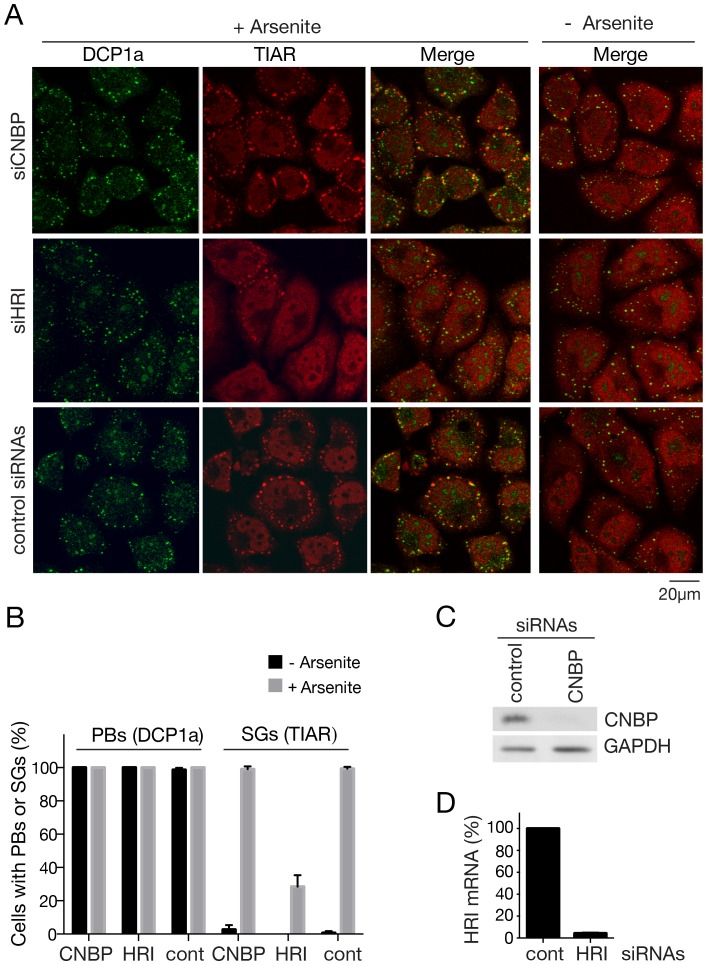
Depletion of CNBP does not affect stress granule formation. (A) siRNAs against CNBP, HRI or nontarget siRNAs were transfected into HeLa cells. After 72 hours, cells were subjected to immunofluorescence to detect DCP1a and TIAR. (B) Histogram showing the percentage of cells with P-bodies and stress granules before and after arsenite induction. P-bodies and stress granules were visualized by performing immunofluorescence with anti-DCP1a and anti-TIAR antibodies, respectively. Data are from three independent experiments. (C) The efficiency of CNBP depletion was monitored by Western blotting. GAPDH was used as a loading control. (D) The efficiency of HRI mRNA depletion was assayed using quantitative RT-PCR.

To determine if CNBP is important for stress granule assembly or integrity, we used siRNAs to reduce CNBP levels. As a positive control, we also depleted heme regulated inhibitor (HRI), which is important for stress granule assembly [58]. Following incubation of the cells with arsenite, the fraction of HRI-depleted cells containing stress granules was strongly reduced, as measured by immunofluoresence to detect TIAR and the translation initiation factor eIF3, although the levels of P-bodies (detected with anti-DCP1a antibodies) were unchanged (Figures 9A, 9B and S3). However, CNBP-depleted cells contained similar levels of both stress granules and P-bodies as cells receiving control non-targeting siRNAs (Figures 9A and 9B). Western blotting revealed that at the time examined (72 hours after transfection), CNBP levels were reduced by 96% (Figure 9C). Thus, although CNBP accumulates strongly in stress granules upon arsenite incubation, it does not appear to be required for stress granule assembly.

## Discussion

Despite the possible involvement of CNBP in DM2 pathogenesis, the cellular functions of this protein and its orthologs are not well understood. Our experiments demonstrate that *S. cerevisiae* Gis2 interacts with translation initiation factors and is a novel component of P-bodies and stress granules. Consistent with a conserved function, CNBP associates with the poly(A) binding protein and accumulates in stress granules during arsenite treatment. These observations implicate both Gis2 and CNBP in mRNA metabolism during stress and should facilitate future studies that define the precise molecular mechanisms by which these proteins act.

Taken together, our results are consistent with a model in which Gis2 and CNBP function at the level of translation initiation to influence mRNA translation during stress. The finding that Gis2 displays RNA-dependent interactions with Pab1 and both eIF4G isoforms suggests that Gis2 may bind mRNAs that are undergoing circularization to form closed loop mRNPs. In support of an interaction at this step in initiation, the cap-binding protein eIF4E is also present in our Gis2-TAP purification (Table S1), and a recent study identified nearly 1000 Gis2-associated mRNAs [27]. Our discovery that Gis2 and CNBP are components of stress granules, which contain mRNPs stalled at a step prior to 60S subunit joining [39], [40], both supports the idea that these proteins interact with mRNAs during initiation and suggests that Gis2 and CNBP could contribute to the translational repression of at least some mRNAs during stress. Although not definitive, our finding that polyribosome levels are slightly increased in *gis2*Δ *dhh1*Δ cells during glucose deprivation (Figure 5F) also supports the hypothesis that Gis2 contributes to translational repression during stress.

Several recent studies have also suggested roles for Gis2 and CNBP in mRNA metabolism. In one set of studies, CNBP was isolated on an affinity column containing RNA derived from the internal ribosome entry sequence (IRES) of the ornithine decarboxylase (ODC) mRNA [23]. Because siRNAs against CNBP reduced internal initiation of an IRES-containing reporter in human cells, and overexpression of either CNBP or Gis2 increased translation of the reporter, both CNBP and Gis2 were proposed to function in internal initiation [9], [15], [23]. In another study, immunoprecipitation of Gis2-associated mRNAs resulted in the identification of hundreds of potential targets [27]. However, comparisons of mRNA levels in wild-type and *gis2*Δ cells, and of mRNA and protein levels in *GIS2*-overexpressing cells, did not reveal simple correlations between Gis2 levels and the fate of these mRNAs [27]. Although our data do not address whether Gis2 functions in internal initiation, our results that Gis2 shows RNA-dependent interactions with translation initiation factors and is a component of P-bodies and stress granules are consistent with both the finding that Gis2 associates with mRNAs and the proposal that Gis2 binding may impact mRNA translation and stability [27].

Finally, our finding that human CNBP accumulates in stress granules is notable in light of *in vitro* studies demonstrating that CNBP binds the 5′-terminal oligopyrimidine (5′TOP) tracts that are required for efficient translational repression of many mRNAs encoding ribosomal proteins and translation factors [7], [20]. These 5′TOP mRNAs were recently shown to require TIA-1 and TIAR for their repression and to accumulate in stress granules upon amino acid starvation [59]. Given proposals that CNBP binding could influence the translation of these mRNAs [7], [20], determining whether CNBP cooperates with TIA-1 and TIAR to regulate 5′TOP mRNA translation or whether it plays a more general role in mRNA metabolism could be interesting future directions.

## Materials and Methods

### Yeast Strains, Media and Plasmids

Strains used in this study are derived from S288C and are listed in Table S2. Strains were grown at 30°C on yeast extract/peptone (YEP) or synthetic complete (SC) media supplemented with appropriate amino acids. As a carbon source, 2% dextrose (Glu), galactose (Gal) or sucrose (Suc) was added. To place three copies of FLAG at the Gis2 C-terminus, an integration cassette was amplified from p3FLAG-KanMX [60]. The mCherry protein was fused to the Gis2 C-terminus using plasmid pBS34 (Yeast Resource Center, University of Washington). Mutant alleles of *DHH1* and *PAT1* were created by using PCR to replace nts 1-2313 of the *PAT1* and nts 106-1446 of the *DHH1* coding sequences with *HIS3*. Other strains carrying null alleles were purchased from Open Biosystems (Huntsville, Alabama). The plasmid pRP1189 expressing *EDC1* mRNA under control of the *GAL1* promoter [52] was a gift of R. Parker (U. of Colorado, Boulder). To generate strain MR204 expressing the *MFA2pG* and *PGK1pG* reporters (Table S2), the plasmid pRP484 [50] was integrated into the *CUP1* locus of BY4741.

### Purification of Gis2-TAP and Mass Spectrometry

Yeast cells (*GIS2-TAP* and wild-type BY4741) were grown in YPD to OD_600_ = 2.0, harvested, washed with water, and lysates prepared and subjected to TAP purification as described [61]. Multidimensional Protein Identification Technology (MudPIT) was carried out as described [28].

### Immunoprecipitations from Yeast Extracts

Yeast cells were grown in YPD to OD_600_ = 2.0, washed with water and resuspended in 600 µl of NET-2 (40 mM Tris-HCl pH 7.5, 150 mM NaCl, 0.05% Nonidet P-40) and 0.25 mM phenylmethylsulfonyl fluoride (PMSF). After vortexing with glass beads, extracts were cleared by sedimentation at 20,000×*g* for 20 min at 4°C. *GIS2-GFP* lysates were incubated with monoclonal anti-GFP (Roche Diagnostics) for 1 h at 4°C followed by Protein G Sepharose beads (GE Healthcare) overnight at 4°C. For RNase A treatment, lysates were incubated with the indicated concentrations of DNase-free RNase A (Sigma-Aldrich) for 10 min at 25°C. Proteins in immunoprecipitates were subjected to Western blotting as described [62]. Antibodies were rabbit anti-FLAG (Sigma-Aldrich), mouse monoclonal anti-GFP (Roche Diagnostics), rabbit anti-ribosomal protein L1A/B (gift of J. Woolford, Carnegie Mellon University), mouse monoclonal anti-Pgk1 (Invitrogen), monoclonal anti-Pab1 (a gift of M. Swanson, University of Florida) and rabbit isoform-specific antibodies against eIF4G1 and eIF4G2 [63] (gifts of J. Doudna, University of California, Berkeley).

### Visualization of Yeast P-bodies and Stress Granules

P-bodies and stress granules were detected largely as described [44]. For glucose depletion experiments, yeast cells were grown in YPD to OD_600_ between 0.35 and 0.5, washed, resuspended in fresh YEP containing or lacking 2% glucose and incubated for 10 min at 30°C. Cells were then harvested and resuspended in 50 µl of the same media. After adding an equal volume of 2% low melting point agarose [in Dulbecco’s phosphate buffered saline lacking calcium and magnesium (Gibco)], the mixture was placed on a glass slide and examined immediately. For stationary phase experiments, cells were grown in YPD for four days, harvested, resuspended in 50 µl of the spent media, and mixed with low melting point agarose as above. Images were acquired with a Zeiss LSM510 confocal microscope using the 63X objective with 3X zoom and processed with the accompanying LSM Image software. For colocalization experiments, single confocal plane images were analyzed. For quantification, at least three independent experiments were performed, with 75–100 cells analyzed per replicate.

### Polysome Analyses

Polysome profiles were compared in wild-type, *gis2*Δ , *pat1*Δ , *dhh1*Δ , *gis2*Δ *pat1*Δ and *gis2*Δ *dhh1*Δ yeast cells (Figure 5) largely as described [64]. Briefly, yeast cultures were grown in YPD to OD_600_ = 0.35–0.5, divided in half, pelleted and washed with YEP that either contained or lacked 2% glucose. Cells were resuspended and incubated in the corresponding medium for 10 min, then harvested in chilled bottles in the presence of 100 µg/ml cycloheximide (Sigma, St Louis, MO). After washing once with lysis buffer A [20 mM Tris-HCl, pH 8, 140 mM KCl, 5 mM MgCl_2_, 0.5 mM DTT, 1% Triton X-100, 100 µg/ml cycloheximide, 1 mg/ml heparin], cell pellets were frozen in liquid nitrogen. To prepare extracts, pellets were suspended in 400 µl of lysis buffer and an equal volume of glass beads was added. Cells were lysed by vortexing for 1 min intervals, followed by 1 min incubations on ice. After centrifuging at 2900×*g* for 2 min at 4°C, lysates were overlaid on 12 ml 15–50% sucrose gradients in lysis buffer A lacking Triton X-100 and sedimented for 2.5 h at 39,000 rpm at 4°C in a Beckman SW40 rotor. Gradients were collected with an ISCO (Lincoln, NE) Model 185 density gradient fractionator.

Because little Pab1 sedimented with polyribosomes under the above conditions, possibly because non-ribosomal proteins were removed by the heparin, we examined the association of Gis2, Pab1 and eIF4G1/2 with polysomes as described [65] with minor modifications. Yeast cells (OD_600_ = 0.4) were collected in chilled bottles in the presence or absence of 100 µg/ml cycloheximide, washed with lysis buffer B [20 mM HEPES-KOH pH 7.6, 100 mM potassium acetate, 5 mM magnesium acetate, 1 mM DTT, 0.1 mM PMSF, 1X EDTA-free protease inhibitor cocktail (Roche) in the presence or absence of 100 µg/ml cycloheximide], harvested, resuspended in lysis buffer B and disrupted by vortexing with glass beads as described above. After sedimenting for 10 min at 6000×*g* at 4°C, cleared lysates were overlaid on 12 ml 15–50% sucrose gradients prepared in lysis buffer B. Micrococcal nuclease treatment of extracts was carried out as described [66].

To examine the sedimentation of CNBP with polysomes, HeLa cell lysates were analyzed largely as described [67]. Briefly, 100 µg/ml cycloheximide was added to the culture media for 5 min before harvesting. Cells were washed with PBS containing 100 µg/ml cycloheximide and harvested by scraping into the same media. After sedimenting for 3 min at 500×g, cells were lysed in 1 ml cold lysis buffer [20 mM Tris-HCl, pH 8, 140 mM KCl, 5 mM MgCl2, 1 mM DTT, 0.5% NP-40, 100 µg/ml cycloheximide, 1 U/ml RNase OUT (Invitrogen), 1 mM PMSF, 1× protease inhibitor cocktail tablet (Roche Diagnostics)] using a Teflon-glass homogenizer. After removing cellular debris by centrifuging 1000×*g* for 10 min and 15,000×*g* for 20 min at 4°C, lysate was overlaid on 11 ml 15–50% sucrose gradients in 20 mM Tris-HCl, pH 8, 140 mM KCl, 5 mM MgCl2, 1 mM DTT, 100 µg/ml cycloheximide, 1 mM PMSF and sedimented as described for yeast extracts. Where noted, cells were cultured with 200 µM puromycin (Sigma, St Louis, MO) for 20 min [68] prior to harvesting in cycloheximide as described above.

### [^35^S]-methionine Incorporation Assays

Assays were performed largely as described [45]. Yeast cultures were grown overnight in SC containing 2% glucose without methionine (SCD-Met) to OD_600_ = 0.4, and two 8-ml aliquots were pelleted by centrifuging at 1300×*g* for 3 min at 4°C. Cells were resuspended in 8 ml of either SCD-Met or SC-Met. After a 20 min incubation with shaking at 30°C, methionine was added to a final concentration of 60 ng/ml, of which 0.5 ng/ml was [^35^S]-methionine (Perkin Elmer). At intervals, 1 ml aliquots were removed, mixed with 125 µl of 100% trichloroacetic acid (TCA), heated to 95°C for 20 min and collected on GFC filters (Whatman). After washing with 10% TCA and 95% ethanol, filters were dried and placed in scintillation fluid (Opti-Fluor, Perkin Elmer) and quantified by scintillation counting. The [^35^S] methionine incorporation rate was determined as described [49]. Each assay was performed at least three times.

### Yeast Transcriptional Shut-off Experiments

Transcriptional shut-off experiments were performed as described [49]. Briefly, yeast cells were grown in appropriate media containing 2% galactose to OD_600_ = 0.3–0.5. Cells were harvested by sedimenting for 3 min at 2900×*g* at 4°C and resuspended in media containing 4% dextrose. At intervals, aliquots were collected and the cells pelleted and frozen on dry ice. RNA was extracted with hot acid phenol as described [69] and fractionated in 5% polyacrylamide/8.3 M urea gels or 1.5% formaldehyde agarose gels and transferred to Hybond N (G.E. Healthcare). Northern blots were probed with [γ-^32^P]ATP-labeled oligonucleotides as described [70]. The oligonucleotides that detect MFA2pG (oRP140), PGK1pG (oRP141) and EDC1 (oRP1121) were described [71]. The oligonucleotide used to detect SCR1 was 5′-TCAACGTATCCCATCCCAC-3′. Data was quantified using a Storm 860 Molecular Dynamics (Sunnyvale, CA) phosphorimager.

### HeLa Cell Culture and Transfections

HeLa cells (a gift of P. De Camilli, Yale University) were maintained in DMEM (Invitrogen) supplemented with 10% fetal bovine serum. Cells at ∼60% confluency were transfected with 4 µg DNA using Lipofectamine 2000 (Invitrogen). The mRFP-DCP1a and mRFP-RCK plasmids were gifts of Nancy Kedersha (Brigham and Women’s Hospital, Harvard Medical School). For RNAi experiments, cells were transfected with 40 nM siRNA (final concentration) using Lipofectamine 2000. After 24 h, cells were trypsinized, seeded onto coverslips and cultured for another 48 h. The siRNAs used were CNBP, siGENOME SMARTpool (Thermo Scientific), HRI (EIF2AK1) [58]: GAAGUACACCACCAAUUUA (Applied Biosystems) and control U0 siRNA [58]: GAAUGCUCAUGUUGAAUCA (Applied Biosystems). Knockdown efficiency was confirmed by Western blotting (CNBP) or quantitative real-time PCR (HRI). To monitor HRI mRNA depletion, the primers 5′-ACACCAACACATACGTCCAG-3′ (forward) and 5′-GCTCCATTTCTGTTCCAAACG-3′ (reverse) were used in quantitative real-time PCR with the control ß-actin primers 5′-ATCAAGATCATTGCTCCTCCTGAG-3′ (forward) and 5′-CTGCTTGCTGATCCACATCTG-3′ (reverse). Differential mRNA expression was measured using the Δ Δ Ct method.

### Immunofluorescence, Immunoprecipitation and Immunoblotting Experiments Using HeLa Cells

Immunofluorescence was carried out as described [72]. To induce stress granule formation, cells were cultured for 30 min with sodium arsenite (0.5 mM). To examine the effects of cycloheximide, the arsenite-containing media was removed, and fresh media added containing either no cycloheximide (control) or 10 µg/ml cycloheximide as described [57]. Images were acquired with a Zeiss LSM510 confocal microscope using the 63X objective and processed with ImageJ software. For co-localization, images were collapsed from 8 z-sections. Immunoprecipitations were carried out largely as described [73]. Briefly, cells were harvested, washed in phosphate-buffered saline (PBS) and sonicated in NET-2 (40 mM Tris-HCl pH 7.5, 150 mM NaCl, 0.05% Nonidet P-40 Alternative (Calbiochem) containing 1 mM PMSF and 1X protease inhibitor cocktail (Roche Diagnostics). After sedimenting twice at 16,000×*g* for 15 min at 4°C, lysates were incubated with anti-CNBP antibody or nonimmune rabbit serum conjugated to protein A-Sepharose (GE Healthcare). After incubating for 1.5 h at 4°C, the beads were washed four times with NET2. Bound proteins were eluted by boiling in SDS-PAGE sample buffer. Antibodies used were rabbit anti-human CNBP (gift of M. Paul Murphy, University of Kentucky), mouse anti-TIAR (BD Transduction Laboratories), rabbit anti-DCP1a (gift of J. Lykke-Andersen, University of California, San Diego), goat anti-eIF3η (N-20) and mouse anti-RPS6 (both Santa Cruz Biotechnology), mouse anti-PABPC1 (gift of G. Dreyfuss, University of Pennsylvania), rabbit anti-eIF4G1 (gift of R. Schneider, New York University School of Medicine, rabbit anti-eIF4G2 (gift of N. Sonenberg, McGill University) and mouse anti-GAPDH (Sigma-Aldrich). Secondary antibodies used in immunofluorescence were goat anti-rabbit IgG and donkey anti-rabbit IgG conjugated to Alexa-fluor 488 and goat anti-mouse IgG and donkey anti-goat IgG conjugated to Alexa-fluor 594 (all Molecular Probes).

## Supporting Information

Figure S1 **Translation rates during glucose depletion.** (A–D). The incorporation of [^35^S]-methionine into protein during growth in glucose (black squares) and during incubation in media lacking glucose (white squares) was measured in (A) wild-type, (B) *gis2*Δ, (C) *dhh1*Δ and (D) *gis2*Δ *dhh1*Δ cells. Each datapoint represents the mean from three independent experiments. For each strain, the percent decrease in the rate of [^35^S]-methionine incorporation after the shift to media lacking glucose was calculated by separately plotting the values from each of the three independent trials and using a best-fit line to measure the slope in the linear range [49].
(TIF)

Figure S2 **Gis2 is not required for efficient decay of** ***MFA2pG*** **,** ***PGK1pG*** **and** ***EDC1*** **mRNAs.** (A–C). Transcriptional shut-off experiments were performed to compare the steady state half-lives of (A) *MFA2pG*, (B) *PGK1pG* and (C) *EDC1* mRNAs in wild-type and *gis2*Δ cells. Following growth in the presence of galactose, cells expressing the indicated reporters were harvested and resuspended in glucose-containing media to repress transcription. At intervals, cells were collected and RNA extracted and subjected to Northern analyses. As a loading control, blots were reprobed to detect the signal recognition particle RNA scR1. Three independent experiments were performed, and mRNA half-lives calculated as described [51]. For each set, a single representative experiment is shown. (D). Transcriptional shut-off analyses were performed as in (A–C) to compare the decay of the *EDC1* mRNA reporter in *dhh1*Δ and *gis2*Δ *dhh1*Δ cells.
(TIF)

Figure S3 **CNBP depletion does not alter the accumulation of eIF3 in stress granules.** (A) After 72 hours, HeLa cells transfected with siRNAs against CNBP, HRI or nontarget siRNAs were subjected to immunofluorescence to detect DCP1a and eIF3η. (B) Histogram showing the fraction of cells with P-bodies (visualized with anti-DCP1a) and stress granules (visualized with anti- eIF3η) before and after arsenite induction. Data are from three independent experiments.
(TIF)

Table S1 **Proteins identified by MUDPIT in Gis2-TAP eluates.**
(PDF)

Table S2 **Yeast strains used in this study.**
(PDF)
