# Supplementary figures and images for: Yeast Gis2 and Its Human Ortholog CNBP Are Novel Components of Stress-Induced RNP Granules
Source: PLoS One. 2012 Dec 21;7(12):e52824. doi: 10.1371/journal.pone.0052824 (PMC3528734; doi:10.1371/journal.pone.0052824)

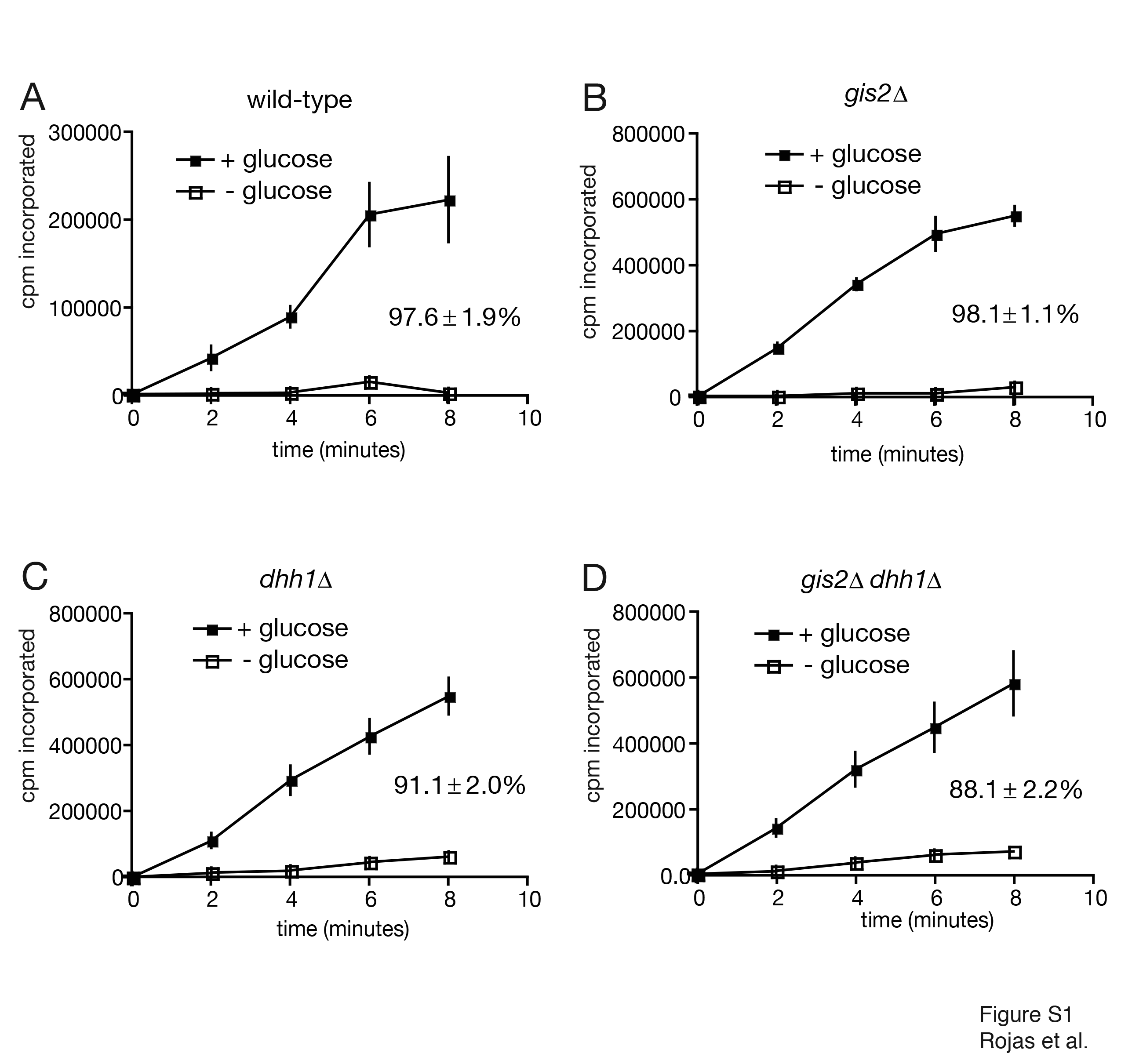

Supplement: Figure S1 — Translation rates during glucose depletion. (A–D). The incorporation of [35S]-methionine into protein during growth in glucose (black squares) and during incubation in media lacking glucose (white squares) was measured in (A) wild-type, (B) gis2Δ, (C) dhh1Δ and (D) gis2Δ dhh1Δ cells. Each datapoint represents the mean from three independent experiments. For each strain, the percent decrease in the rate of [35S]-methionine incorporation after the shift to media lacking glucose was calculated by separately plotting the values from each of the three independent trials and using a best-fit line to measure the slope in the linear range [49]. (TIF) [file pone.0052824.s001.tif]

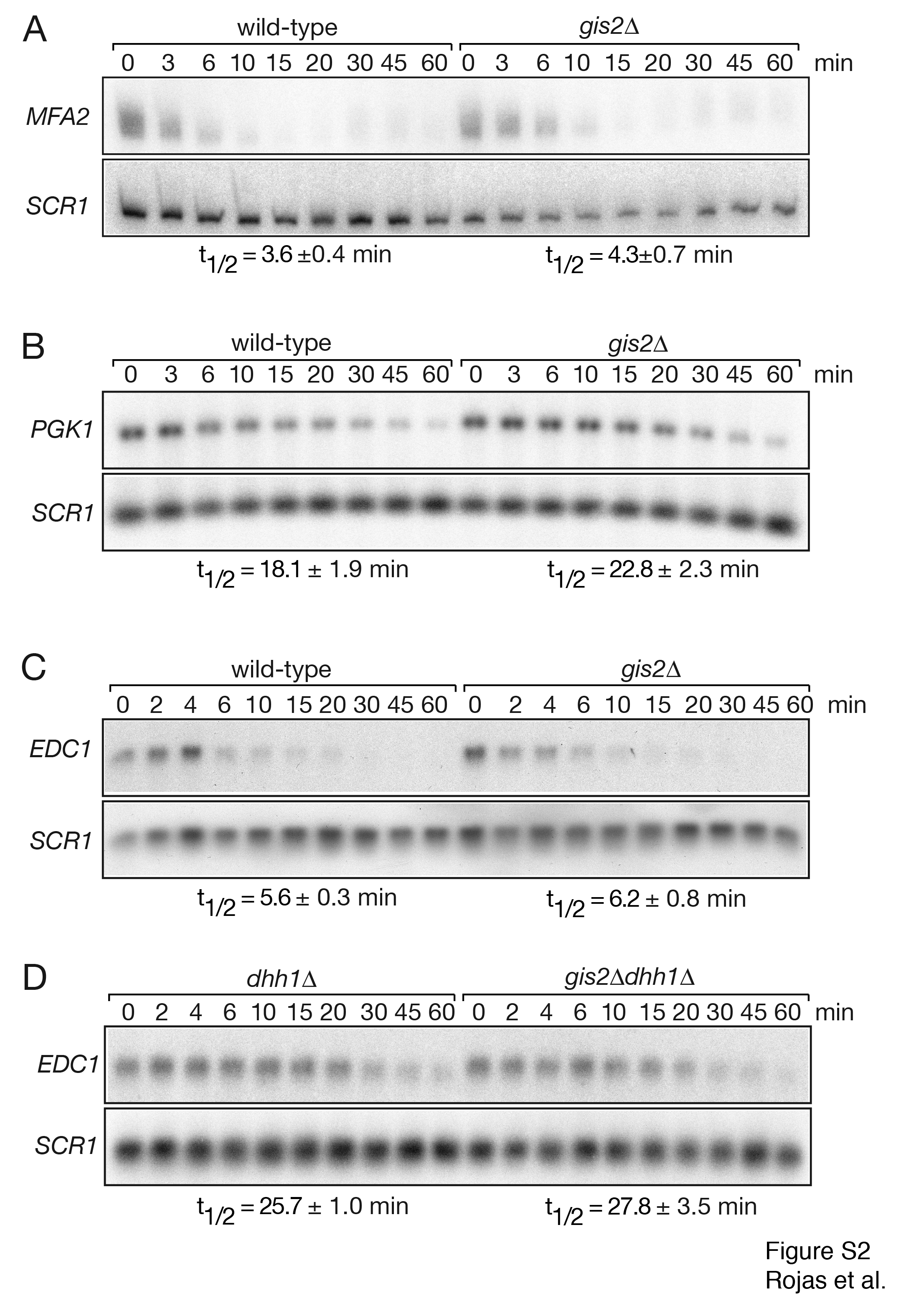

Supplement: Figure S2 — Gis2 is not required for efficient decay of MFA2pG , PGK1pG and EDC1 mRNAs. (A–C). Transcriptional shut-off experiments were performed to compare the steady state half-lives of (A) MFA2pG, (B) PGK1pG and (C) EDC1 mRNAs in wild-type and gis2Δ cells. Following growth in the presence of galactose, cells expressing the indicated reporters were harvested and resuspended in glucose-containing media to repress transcription. At intervals, cells were collected and RNA extracted and subjected to Northern analyses. As a loading control, blots were reprobed to detect the signal recognition particle RNA scR1. Three independent experiments were performed, and mRNA half-lives calculated as described [51]. For each set, a single representative experiment is shown. (D). Transcriptional shut-off analyses were performed as in (A–C) to compare the decay of the EDC1 mRNA reporter in dhh1Δ and gis2Δ dhh1Δ cells. (TIF) [file pone.0052824.s002.tif]

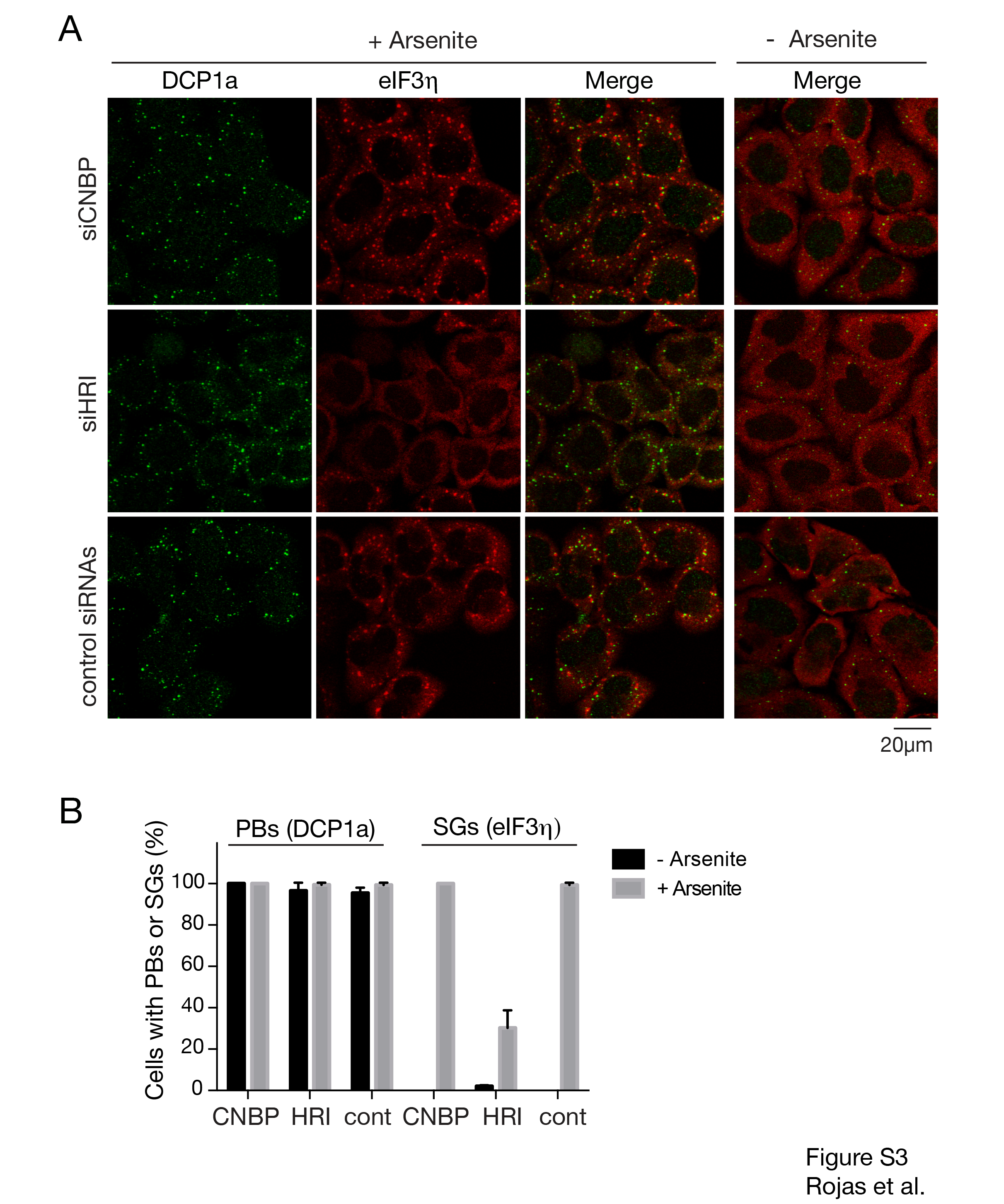

Supplement: Figure S3 — CNBP depletion does not alter the accumulation of eIF3 in stress granules. (A) After 72 hours, HeLa cells transfected with siRNAs against CNBP, HRI or nontarget siRNAs were subjected to immunofluorescence to detect DCP1a and eIF3η. (B) Histogram showing the fraction of cells with P-bodies (visualized with anti-DCP1a) and stress granules (visualized with anti- eIF3η) before and after arsenite induction. Data are from three independent experiments. (TIF) [file pone.0052824.s003.tif]
