# Supplementary material for: Yeast Gis2 and Its Human Ortholog CNBP Are Novel Components of Stress-Induced RNP Granules
Source: PLoS One. 2012 Dec 21;7(12):e52824. doi: 10.1371/journal.pone.0052824 (PMC3528734; doi:10.1371/journal.pone.0052824)
Supplement: Table S1 — Proteins identified by MUDPIT in Gis2-TAP eluates. (PDF) [file pone.0052824.s004.pdf]

Table S1. Proteins identified by MUDPIT in Gis2-TAP eluates

Protein	Spectral Counts	Sequence Coverage (%)
Rpl22A	274	47.1
Rpl4A/B*	245	45.9
Pab1	226	57.9
Ded1*	183	57.8
Tdh3*	173	54.5
Tdh2	166	45.2
Rps17A/B	136	54.4
RpS7A/B*	98	43.7
Rpl43A/B	98	43.5
eIF4G1	96	38.7
**Gis2**	**94**	**80.4**
Ssb2*	81	52.9
RpS4A/B	81	39.8
Ssb1*	80	52.9
Imd3	79	40.0
Rps16A/B	74	60.1
Xrn1	66	22.5
Ssa2*	65	50.9
Rps19A/B*	64	48.6
Rpl7A/B*	64	41.4
Imd4	63	36.6
Imd2	57	28.7
Ssa1*	55	41.9
Rps13*	53	43.7
eIF4G2	53	35.0
Rpl35A/B	51	31.7
Rpl17A/B*	48	47.8
Rpl13A/B	48	43.7
Rpl8A/B	47	48.8
Rpl3*	45	31.3
Ssd1	44	25.3
Rps1A/B	42	51.0
Rpl20A/B	42	44.2
eIF4E	42	44.1
Rps26A*	39	26.9
Rpl14A/B	38	32.6
Pwp1	38	50.0
Sse1*	38	29.1
Rpl28	38	22.1
Rps25A/B	37	39.8
Rpl26A/B	36	56.0
RpS22A/B	36	46.9

Nop56	36	31.5
Eno2*	34	42.6
Dbp2	34	30.8
Cdc19*	34	30.8
Rps8A/B	31	46.5
Rpl31A/B	39	46.0
Rps2	39	40.2
Rps6A/B*	36	40.7
Rpl16A/B	32	41.7
Rps24A/B*	31	44.4
Eap1	31	37.7
Sro9	31	26.5
Ubp3	31	25.9
Npl3	30	23.2
Rpl15A	29	32.4
Pgk1*	28	45.7
Stm1	28	43.2
Nop58	28	24.1
EF-1α*	28	19.2
Sbp1	27	45.6
Rpp0	27	29.8
Sui3	26	43.5
Gpm1*	26	37.2
Rpl6A/B	25	41.5
Rpl5	25	40.4
Rpl2A/B*	25	39.8
Rpa49	25	32.3
Mis1	25	13.1
Rps5	24	39.1
Rpl19A/B*	23	34.9
Rpl11A/B	23	31.6
Pdc1	23	29.5
Psp1	23	22.1
Rps14A/B*	22	48.9
Adh1	22	26.7
Vip1	22	16.1
Nop1	21	39.4
Rps9A/B	21	34.9
Rps3	20	50.8
Rpl36A/B	20	42.0
Rpl9A/B	20	29.3
Trm1	20	23.3
Pbp1	20	21.7
Rpl10*	19	38.0
Nsr1	19	37.4

Rpl1A/B	19	35.9
Yra1	19	27.9
EF-2 (Eft1/Eft2)	19	16.5
Rpl25	18	47.2
Rps30A/B	18	42.9
Pat1	18	19.5
Syp1	17	14.1
Ura2	16	6.7
Rpl12A/B	15	46.7
Rps23A/B	15	32.4
Rpl18A/B	15	26.3
Nop53	15	17.1
Rrp5	15	8.8

*common contaminant of TAP purifications [29]
