# Supplementary material for: Yeast Gis2 and Its Human Ortholog CNBP Are Novel Components of Stress-Induced RNP Granules
Source: PLoS One. 2012 Dec 21;7(12):e52824. doi: 10.1371/journal.pone.0052824 (PMC3528734; doi:10.1371/journal.pone.0052824)
Supplement: Table S2 — Yeast strains used in this study. (PDF) [file pone.0052824.s005.pdf]

Table S2. Yeast strains used in this study

Strain	Genotype	Source
MR199	*MAT*a *his3Δ1 leu2Δ0 met15Δ0 ura3Δ0*	Invitrogen
MR200	*MAT*a *his3Δ1 leu2Δ0 met15Δ0 ura3Δ1 gis2::KanMX*	Invitrogen
MR243	*MAT*a *his3Δ1 leu2Δ0 met15Δ0 ura3Δ0 dhh1::HIS3*	This study
MR242	*MATα his3Δ1 leu2Δ0 met15Δ0 ura3Δ0 gis2::KanMX dhh1::HIS3*	This study
MR244	*MATα his3Δ1 leu2Δ0 lys2Δ0 ura3Δ0 pat1::HIS3*	This study
MR247	*MAT*a *his3Δ1 leu2Δ0 lys2Δ0 ura3Δ0 gis2::KanMX pat1::HIS3*	This study
MR213	*MAT*a *his3Δ1 leu2Δ0 met15Δ0 ura3Δ0 GIS2-TAP(HIS3)*	Open Biosystems
MR233	*MAT*a *his3Δ1 leu2Δ0 met15Δ0 ura3Δ0 GIS2-GFP(HIS3)*	Invitrogen
MR588	*MATα his3Δ1 leu2Δ0 met15Δ0 ura3Δ0 GIS2-FLAG*$_3$*(KanMX) PAB1-GFP(HIS3)*	This study
MR590	*MAT*a *his3Δ1 leu2Δ0 met15Δ0 ura3Δ0 GIS2-FLAG*$_3$*(KanMX) EIF4G1-GFP(HIS3)*	This study
MR592	*MAT*a *his3Δ1 leu2Δ0 met15Δ0 ura3Δ0 GIS2-FLAG*$_3$*(KanMX) EIF4G2-GFP(HIS3)*	This study
MR469	*MATα his3Δ1 leu2Δ0 met15Δ0 ura3Δ0 GIS2-mCherry(KanMX) PUB1-GFP(HIS3)*	This study
MR487	*MATα his3Δ1 leu2Δ0 met15Δ0 ura3Δ0 GIS2-mCherry(KanMX) PAB1-GFP(HIS3)*	This study
MR480	*MAT*a *his3Δ1 leu2Δ0 met15Δ0 ura3Δ0 GIS2-mCherry(KanMX) EIF4G1-GFP(HIS3)*	This study
MR584	*MAT*a *his3Δ1 leu2Δ0 met15Δ0 ura3Δ0 GIS2-mCherry(KanMX) EIF4G2-GFP(HIS3)*	This study
MR684	*MATα his3Δ1 leu2Δ0 met15Δ0 ura3Δ0 GIS2-mCherry(KanMX) DCP2-GFP(HIS3)*	This study
MR687	*MATα his3Δ1 leu2Δ0 met15Δ0 ura3Δ0 GIS2-mCherry(KanMX) EDC3-GFP(HIS3)*	This study
MR204	*MATα his3Δ1 leu2Δ0 met15Δ0 lys2Δ0 ura3Δ0 cup1::LEU2/PGK1pG/MFA2pG*	This study
MR203	*MAT*a *his3Δ1 leu2Δ0 met15Δ0 lys2Δ0 ura3Δ0 gis2::KanMX cup1::LEU2/PGK1pG/MFA2pG*	This study
MR699	*MAT*a *his3Δ1 leu2Δ0 ura3Δ0*	This study
MR708	*MAT*a *his3Δ1 leu2Δ0 ura3Δ0 gis2::KanMX*	This study
MR703	*MAT*a *his3Δ1 leu2Δ0 ura3Δ0 dhh1::HIS3*	This study
MR705	*MAT*a *his3Δ1 leu2Δ0 ura3Δ0 gis2::KanMX dhh1::HIS3*	This study
